# The mTOR Inhibitor Rapamycin Has Limited Acute Anticonvulsant Effects in Mice

**DOI:** 10.1371/journal.pone.0045156

**Published:** 2012-09-12

**Authors:** Adam L. Hartman, Polan Santos, Alison Dolce, J. Marie Hardwick

**Affiliations:** 1 Department of Neurology, Johns Hopkins University School of Medicine, Baltimore, Maryland, United States of America; 2 W. Harry Feinstone Department of Molecular Microbiology and Immunology, Johns Hopkins University Bloomberg School of Public Health, Baltimore, Maryland, United States of America; Max Planck Institute of Psychiatry, Germany

## Abstract

**Objective:**

The mammalian target of rapamycin (mTOR) pathway integrates signals from different nutrient sources, including amino acids and glucose. Compounds that inhibit mTOR kinase activity such as rapamycin and everolimus can suppress seizures in some chronic animal models and in patients with tuberous sclerosis. However, it is not known whether mTOR inhibitors exert acute anticonvulsant effects in addition to their longer term antiepileptogenic effects. To gain insights into how rapamycin suppresses seizures, we investigated the anticonvulsant activity of rapamycin using acute seizure tests in mice.

**Methods:**

Following intraperitoneal injection of rapamycin, normal four-week-old male NIH Swiss mice were evaluated for susceptibility to a battery of acute seizure tests similar to those currently used to screen potential therapeutics by the US NIH Anticonvulsant Screening Program. To assess the short term effects of rapamycin, mice were seizure tested in ≤6 hours of a single dose of rapamycin, and for longer term effects of rapamycin, mice were tested after 3 or more daily doses of rapamycin.

**Results:**

The only seizure test where short-term rapamycin treatment protected mice was against tonic hindlimb extension in the MES threshold test, though this protection waned with longer rapamycin treatment. Longer term rapamycin treatment protected against kainic acid-induced seizure activity, but only at late times after seizure onset. Rapamycin was not protective in the 6 Hz or PTZ seizure tests after short or longer rapamycin treatment times. In contrast to other metabolism-based therapies that protect in acute seizure tests, rapamycin has limited acute anticonvulsant effects in normal mice.

**Significance:**

The efficacy of rapamycin as an acute anticonvulsant agent may be limited. Furthermore, the combined pattern of acute seizure test results places rapamycin in a third category distinct from both fasting and the ketogenic diet, and which is more similar to drugs acting on sodium channels.

## Introduction

Epilepsy affects 0.5–1% of the US population but ∼20–30% of patients do not respond to the two initial medications prescribed [1], [2], [3], [4]. One underutilized option for this population is metabolism-based therapy through dietary or pharmacologic interventions, particularly if the patient does not have a surgically resectable lesion [5]. The most commonly used metabolism-based therapy is the high-fat, low carbohydrate ketogenic diet. The efficacy of the ketogenic diet in children was shown in a randomized controlled trial showing a robust 75% decrease in patient seizures over three months [6]. Small molecules that potentially target the same pathways are being investigated for antiseizure effects, including agents that act on nutrient-sensing mechanisms such as the mTOR-containing TORC1 complex. In cell culture models, depletion of glucose and specific amino acids suppresses mTOR serine-threonine kinase activity, leading to reduced protein translation and induction of autophagy [7]. Mutations in TSC1/2, genes that normally suppress mTOR, are responsible for tuberous sclerosis complex, which includes seizures, tubers, subependymal giant cell tumors, autism, behavior problems, and other systemic complications [8]. In Tsc1- or Pten-deficient mice that have increased mTOR activity and chronic spontaneous seizures, sustained treatment with the mTOR inhibitor rapamycin decreased seizure frequency [9], [10], [11]. Furthermore, the rapamycin analog everolimus restricted tumor growth and decreased seizure frequency in a clinical trial of patients with tuberous sclerosis complex [12].

Inhibitors of mTOR may improve seizure control in other chronic epilepsy models where the underlying cause of epilepsy is not due to mutations in the TOR pathway. For example, rapamycin suppressed behavioral spasms in the doxorubicin/lipopolysaccharide/p-chlorophenylalanine model of infantile spasms [13]. Rapamycin also decreased susceptibility to kainic acid-induced seizures in P13 rats exposed to graded hypoxia at P10 [14]. In addition, rapamycin protected against spontaneous seizures that recur for several months following one-time kainic acid- or pilocarpine-induced status epilepticus in rats [15], [16]. Collectively, these reports with chronic models support the general opinion that rapamycin protects by inducing long-term cellular changes [17]. Rapamycin also protected against seizures when administered after the initial induction of status epilepticus in the pilocarpine rat model [16], raising the possibility that rapamycin also may act acutely to inhibit seizure activity [18]. However, rapamycin failed to protect when the same post-treatment model of pilocarpine-induced status epilepticus was applied to mice [19] and it did not protect against seizures during the first 48 hours after a hypoxic insult in P10 rats, challenging the idea that rapamycin has acute antiseizure effects. Similarly, attempts to study the short-term effects of rapamycin *in vitro* also have not provided strong support for acute effects of rapamycin. Short-term exposure of neurons *in vitro* to rapamycin did not alter neuronal firing under baseline conditions, and it had limited benefits under conditions of provoked neuronal firing [20], [21]. One way to determine if rapamycin acutely suppresses seizure activity is to compare it to known anticonvulsants.

Rapamycin has not been systematically tested in a battery of acute seizure tests like those used routinely to screen candidate therapeutics in preclinical trials [22]. Using similar tests, we found that rapamycin has a limited acute anticonvulsant effect. Furthermore, rapamycin exposure for ≤6 h (defined here as a ‘short’ exposure) has a profile (i.e., a combination of positive and negative seizure test results) that is comparable to drugs that suppress voltage-gated sodium channel activity. Even when tested for longer times (3–13 days), rapamycin still has an acute seizure test profile that does not match the profiles of either the ketogenic diet or another dietary antiseizure intervention, intermittent fasting. Thus, the anticonvulsant mechanisms of rapamycin may be distinct from other metabolism-based therapies. Because of the adverse effects of rapamycin and related drugs in patients [23], finding an explanation for how mTOR inhibition protects against seizures could help facilitate the design of more specific agents and minimize side effects.

## Methods

### Animals

Male NIH Swiss mice (NCI, Frederick, MD, U.S.A.) aged 3–4 weeks were acclimatized to the animal care facility for 1–5 days and housed four per cage. All mice were fed unrestricted normal rodent chow (Teklad Global 2018SX, Madison, WI, U.S.A.). Animals were not fasted except where indicated, when food was withdrawn 24 h prior to the first dose of rapamycin.

### Ethics Statement

This study was carried out in strict accordance with the recommendations in the Guide for the Care and Use of Laboratory Animals of the National Institutes of Health. The protocol was approved by the Johns Hopkins Animal Care and Use Committee (Protocol # MO10M254). All animal protocols are approved by the Animal Care and Use Committee at Johns Hopkins Medical Institutions.

### Rapamycin administration

At 4 weeks of age, mice were injected intraperitoneally with rapamycin 4.5 mg/kg or 6 mg/kg body weight (LC Laboratories, Woburn, MA, U.S.A.) prepared from a stock solution (20 mg/ml in 100% ethanol, stored at −20°C) diluted to a final concentration of 4% (v/v) ethanol in the vehicle immediately prior to use. Vehicle consisted of 5% polyethylene glycol 400 (Sigma, St. Louis, MO, U.S.A.) and 5% Tween 80 (Sigma, St. Louis, MO, U.S.A.) [24]. Both rapamycin and vehicle-treated mice received the same volume of ethanol. To determine the effect of brief rapamycin exposures, we performed seizure tests after ≤6 h of exposure. Longer rapamycin exposures (i.e., ≥3 d) were used to facilitate comparisons with dietary treatments such as the ketogenic diet and intermittent fasting. Mice treated with rapamycin or vehicle for 13 days received injections of rapamycin (4 mg/kg) or vehicle at 48 h intervals starting at 3 weeks of age.

### mTOR activity

Relative levels of mTOR activity were assessed from immunoblots comparing phosphorylated and total levels of ribosomal S6 protein. Tissues were analyzed 0.5 h, 1 h, 3 h, and 6 h after a single rapamycin injection, and after 3 d (∼75 h) of daily rapamycin injections. Mice were sacrificed by cervical dislocation and rapidly dissected neocortex and hippocampi were immediately frozen. Lysates of frozen tissues were prepared with RIPA buffer (NaCl 150 mM, Triton X-100 1%, sodium deoxycholate 0.5%, SDS 0.1%, Tris 50 mM (pH 8.0)) and separated by SDS-PAGE (12%). Blots were probed overnight with antibodies against S6 and phospho-S6 (Ser240/244) (Cell Signaling, Danvers, MA, U.S.A., 1∶500), and for 2 h with horseradish peroxidase-conjugated anti-rabbit secondary antibody (Cell Signaling, Danvers, MA, U.S.A., 1∶500, 1∶10,000). Protein bands were visualized with ECL reagent (Pierce, Thermo Fisher, Rockford, IL, U.S.A.). Band intensity was quantified using ImageJ software (NIH).

### Seizure tests

Each mouse was tested for seizures only once. Personnel performing seizure testing and assessments were blinded to treatment group assignment. Except where indicated, seizure tests were performed at the indicated number of hours following a single dose of rapamycin (4.5 mg/kg), or mice were tested 3 h after receiving three doses of rapamycin (4.5 mg/kg) given at 24 h intervals (75 h).

#### MES-T test

The maximal electroshock threshold (MES-T) test was performed as described previously [25]. Briefly, after pretreatment with tetracaine 0.5% ophthalmic solution (Bausch & Lomb, Tampa, FL, U.S.A.), current was delivered using a Rodent Shocker 221 (Harvard apparatus, Holliston MA, U.S.A.) with a shock duration of 0.2 sec. Currents were selected based on results of responses in serial testing (i.e., a staircase-type protocol). The primary outcome was the presence (or absence) of hindlimb extension to 180 degrees in a rostral-caudal plane. Additional secondary outcomes were seizure duration and seizure scores based on a Racine-type scale that reflected behaviors we observed consistently in progression: 0, no seizure behavior; 1, immobility; 2, wobbling gait; 3, unilateral pawing ± Straub tail; 4, multiple limb clonus; 5, emprosthotonos; 6, tonic hindlimb extension.

#### 6 Hz test

The 6 Hz test was administered using the same apparatus, stimulus frequency (6 Hz), pulse width (0.2 msec), and shock duration (3 sec) as described previously [25]. The primary outcome was the occurrence of seizures, defined as any abnormal activity of any duration, typically including clonus followed by immobility, facial muscle twitching, staring, automatisms including chewing and unilateral pawing, and a Straub tail.

#### Kainic acid test

Kainic acid was injected intraperitoneally (23.5 mg kainic acid/kg mouse body mass, 5.3 mg/ml PBS, Tocris Bioscience, Ellisville, MO, U.S.A.) as described previously [25]. Mice were observed continuously in Plexiglass cages for the duration of the experiment. Seizure behaviors were scored for 2 h using a modified Racine scale (the highest score in a given 5-min block was used): 0, no seizure; 1, immobility; 2, forelimb and/or tail extension; 3, automatisms; 4, forelimb clonus, rearing, and/or falling; 5, repetition of stage 4; 6, tonic–clonic seizures; and 7, death [25], [26]. As the mouse behaviors only occur intermittently, the highest seizure score achieved in a 5 min epoch was recorded.

#### PTZ test

PTZ (10 mg/ml PBS, Tocris Bioscience, Ellisville, MO, U.S.A.) was infused at a constant rate (0.05 ml/min) as reported previously [25]. The outcome threshold dose was calculated as the time to reach each clinical endpoint. Mice were observed continuously for the duration of the infusion. Nearly all mice progressed through a predictable and reproducible series of behaviors: first twitch (sudden tail flick), initial clonus (clonus sustained >10 sec), alternating sustained clonus and immobility, terminal clonus (last episode of clonus prior to tonic posturing), and tonic hindlimb extension (final behavior followed by death, when infusion was terminated). The latter was identical in appearance to the primary endpoint in the MES-T test described previously.

### Glucose and β-hydroxybutyrate testing

One animal per cage selected with a random number generator was used to measure glucose and the ketone body β-hydroxybutyrate (Precision Xtra system; Abbott Laboratories, North Chicago, IL, U.S.A.) as previously reported [25].

### Statistics

Probit analyses (used in the MES-T test to determine the current where half the mice experienced THLE, or THLE_50_ and the current where half the mice experienced any seizure behavior in the 6 Hz test, or CC_50_) were performed using Minitab 16 (State College, PA, U.S.A.). *t*-tests, Mann-Whitney U tests, and nonlinear curve fits were performed using GraphPad Prism 4 (LaJolla, CA, U.S.A.).

## Results

The Anticonvulsant Screening Project funded by the National Institute of Neurological Disease and Stroke (NINDS) screens dozens of potential compounds annually for potential therapeutic use [22]. By adjusting dosing parameters, we adapted these tests to permit direct comparisons with metabolism-based interventions such as the ketogenic diet and intermittent fasting [25]. Using these acute seizure tests, we investigated the possibility that rapamycin has anticonvulsant effects.

### Time course of rapamycin-suppressed mTOR activity

**Figure 1:**
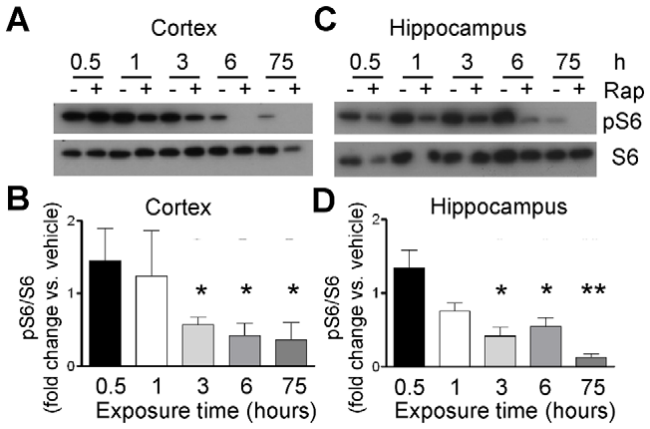
Rapamycin decreases phosphorylation of S6. Immunoblots of phosphorylated and total S6 in dissected brain cortex (**A**) and hippocampus (**B**) are shown at the indicated times after treatment with rapamycin or vehicle. (**C**) Summary data for cortex are indicated as mean+/-SEM of the fold change in pS6 to S6 ratios measured by densitometry (N = 2 mice in 3 independent experiments at 0.5 h, 1 h, and 3 h; N = 8 at 6 h and 75 h). (**D**) Summary data for hippocampus as described for cortex (N = 2 mice in 3 independent experiments at 0.5 h, 1 h, 3 h, and 6 h; N = 10 for 75 h). * p<0.04; ** p  =  0.00003. Each dose of rapamycin was 4.5 mg/kg.

As a first approximation of timing for seizure testing, the effect of rapamycin on mTOR activity in mouse brains was evaluated by assessing the phosphorylation status of ribosomal protein S6, a known downstream target of mTOR [27]. Phospho-S6 (pS6) levels decline shortly after rapamycin treatment compared to vehicle in both cortex (Fig. 1A and B) and hippocampus (Fig. 1C and D). pS6 was dramatically suppressed at 3 h and 6 h after one treatment with rapamycin (4.5 mg/kg), and after three consecutive daily doses of rapamycin (4.5 mg/kg), consistent with a previous report [15].

### Rapamycin transiently protects against seizures in the MES-T test

**Figure 2:**
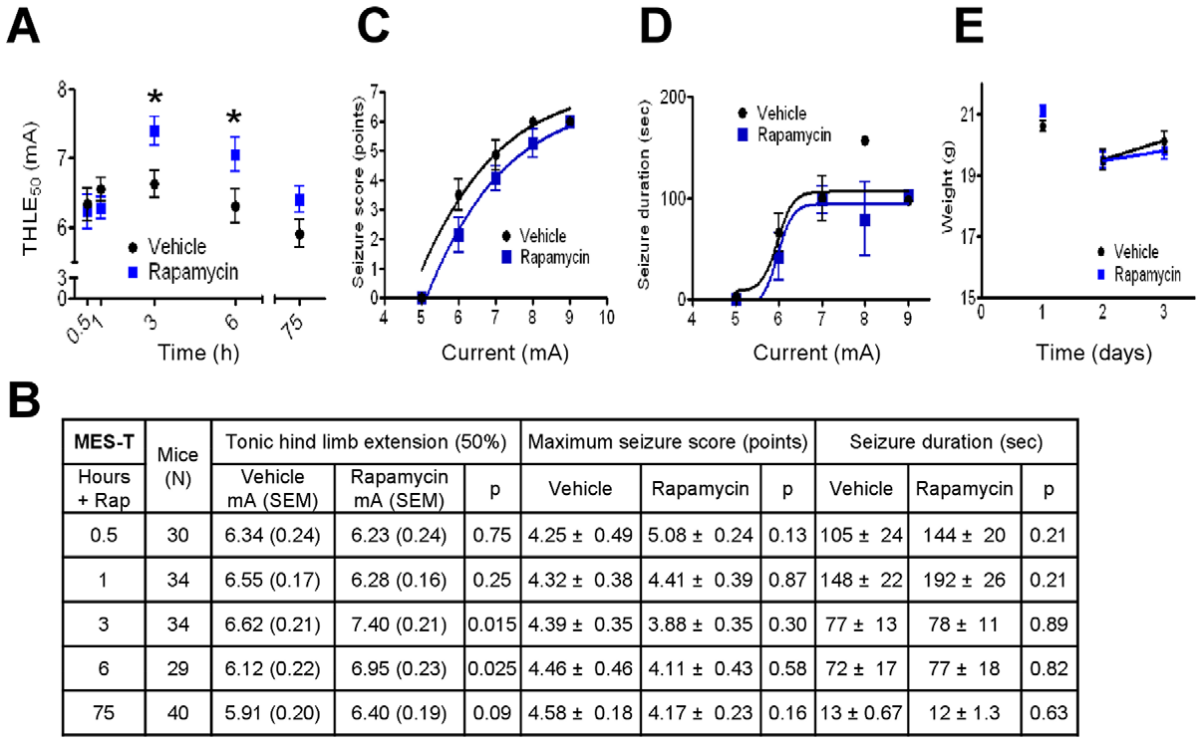
Rapamycin (4.5 **mg/kg) protects transiently against seizures in the maximal electroshock threshold (MES-T) test.** (**A**) Currents where 50% of mice had tonic hindlimb extension (THLE_50_), evaluated using a probit analysis. Data are presented from 3 independent experiments per condition, showing the mean +/- SEM. Numerical values are shown in B. (**B**) Data for all animals tested for THLE (shown graphically in panel A) (all mice), for maximum seizure scores (scale 0–6, where 6 = THLE) (all mice), and for seizure duration, which was measured for all mice that survived (N = 20–31 for vehicle; N = 24–35 for rapamycin per treatment time). Data presented are from 3 independent experiments per condition. Statistical comparisons for maximum seizure score and duration were compared using a *t*-test. (**C**) Maximum seizure score for animals treated for 3 h in panel A using a nonlinear curve fit. (**D**) Seizure duration for the same animals in panel C using a nonlinear curve fit. (**E**) Weights of all mice undergoing the MES-T test, comparing vehicle and rapamycin. Animal cohorts tested at the 0.5, 1, 3, and 6 h time points happen to weigh slightly more than those tested for 3 days, giving the false appearance of weight loss after one day. All mice gained weight through the duration of the experiment. Weights for the same animals weighed at different times are connected by lines.

Mice were protected against the traditional outcome in the MES-T test, tonic hindlimb extension (THLE), at 3 h and 6 h after rapamycin injection, but not at earlier time points (Fig. 2A and B). Despite profound suppression of mTOR, there was only a trend toward protection that was not statistically significant after 3 days of consecutive rapamycin dosing (Fig. 2A and B). To detect more subtle differences in seizure phenotypes, seizure behaviors were scored using a Racine-type scale and the maximum seizure score obtained for each animal was compiled. In addition, seizure duration was measured. Again, there were no differences with rapamycin in either parameter (Fig. 2B). A relationship between maximum seizure score and/or duration might be more notable at each specific stimulus current (i.e., not revealed by the grouped data), so a nonlinear curve fit was performed. However, this analysis showed no additional differences and was less revealing than traditional outputs even at 3 h, when rapamycin had its greatest effect (Fig. 2C & D and data not shown). No differences in body weights were detected between mice treated with rapamycin versus vehicle, as expected (Fig. 2E).

### Rapamycin does not protect against 6 Hz-induced seizures

**Figure 3:**
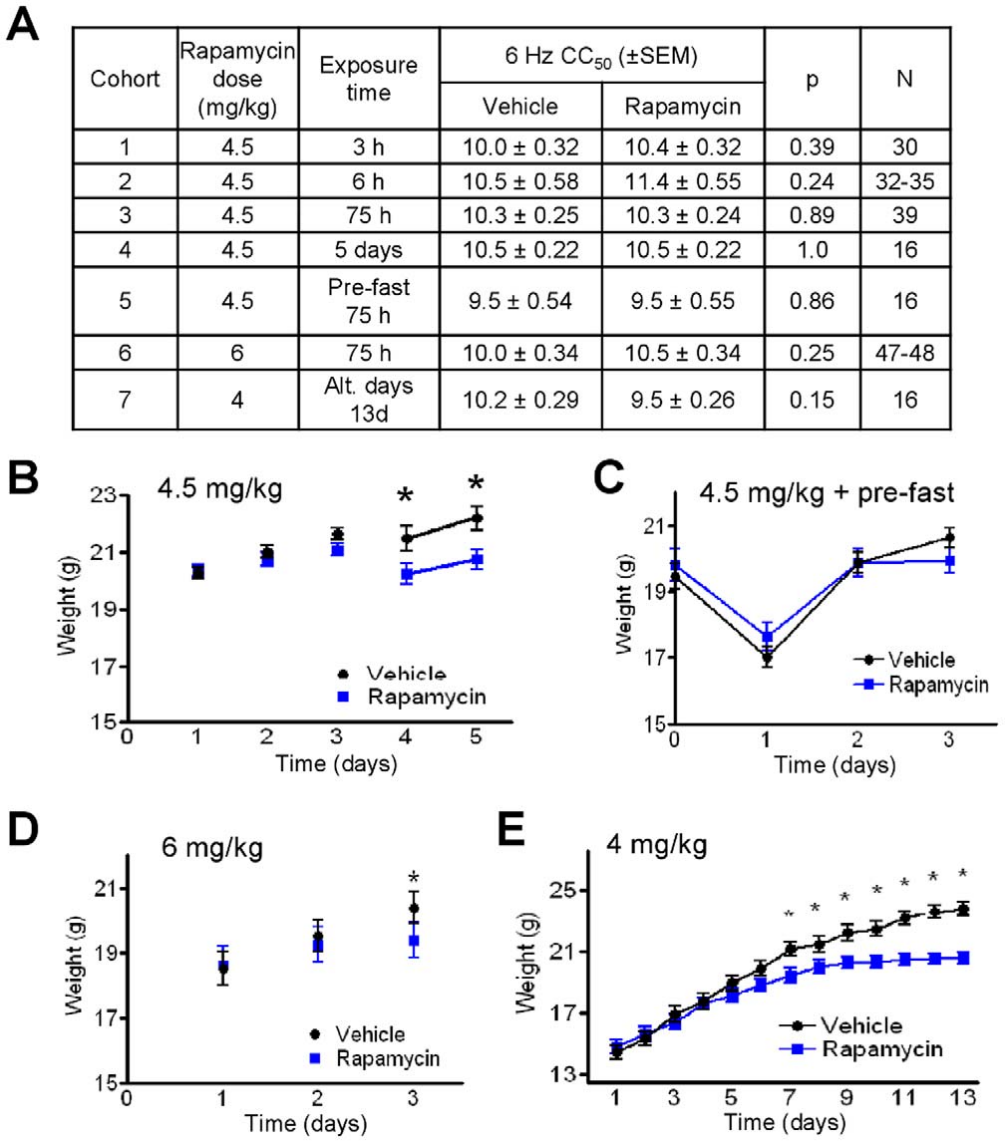
Rapamycin does not protect against 6 Hz-induced seizures. (**A**) Mean convulsion currents (CC_50_ ±SEM), the current at which 50% of mice had convulsions induced by a 6 Hz stimulus after treatment with rapamycin or vehicle at the indicated doses and schedules. Mice in cohorts 1, 2, 3, and 6 were tested in 3 independent experiments for each treatment regimen; mice in cohorts 4, 5, and 7 were tested in a single experiment each. CC_50_ was calculated using a probit analysis. Weights are shown for mice undergoing the 6 Hz test in cohorts 1–4 (**B**), cohort 5 (**C**), cohort 6 (**D**), and cohort 7 (**E**). *, p<0.05 (*t*-test). Weights for the same animals are connected by lines.

The 6 Hz test reveals the protective effects of the ketogenic diet in mice [28]. However, mice were not protected against 6 Hz seizures by treating with rapamycin for 3 h or 6 h, and were not protected by 3 daily doses of rapamycin, even when preceded by an initial fast, or by higher doses of rapamycin (6 mg/kg) (Fig. 3A). To further test rapamycin dosing that might mimic the effects of more chronic administration, as with the typical 12–14 day ketogenic diet [25], the 6 Hz test was performed after 5 consecutive days of rapamycin treatment (4.5 mg/kg, without an initial fast) and after intermittent treatment for 13 days (4 mg/kg, Fig. 3A). However, there was no significant protection by rapamycin even though mice had lower body weights similar to those treated with the ketogenic diet or intermittent fasting (Fig. 3B–E) [25].

### Rapamycin has mixed effects in kainic acid-induced seizures

**Figure 4:**
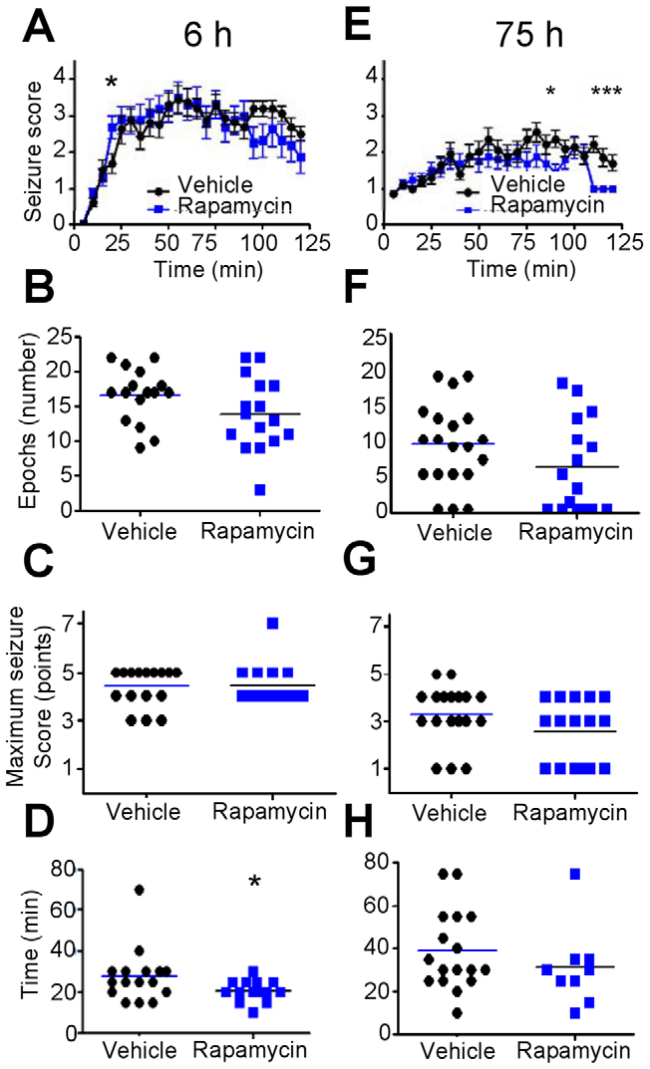
Rapamycin shows varied effects against seizures induced by kainic acid (i.p.). (**A**) Mean seizure scores (±SEM) taken at 5-min intervals for 4 independent cohorts of mice treated with rapamycin (4.5 mg/kg) or vehicle for 6 h (N = 16 mice/group) (p = 0.55). (**B**) Latency to onset of seizure stage ≥2 for mice in panel A (p = 0.04, Mann-Whitney U test; bar represents group mean). (**C**) Number of 5-min time intervals in seizure stage ≥2 for mice in panel A (p = 0.14, Mann-Whitney U test). (**D**) Maximum seizure scores over the entire observation period (2 h) for mice in panel A (p = 0.66, Mann-Whitney U test). (**E**) Mean seizure scores (±SEM) for 3 consecutive days of rapamycin treatment (i.e., 75 h of rapamycin exposure) (N = 16) or vehicle (N = 20) (p = 0.0002; Bonferroni correction for multiple comparisons, p = 0.002). (**F**) Latency to onset of seizure stage ≥2 for mice in panel E (p = 0.31, Mann-Whitney U test). (**G**) Number of 5-min time intervals in seizure stage ≥2 for mice in panel E (p = 0.14, Mann-Whitney U test). (**H**) Maximum seizure scores over the entire observation period (2 h) for mice in panel E (p = 0.15, Mann-Whitney U test). In terms of body weight, there were no statistically significant differences between mice treated with vehicle or rapamycin at 6 h (19.2±0.41 vs. 19.0±0.38, respectively; p = 0.80), 48 h (19.2±0.48 vs. 18.9±0.41, respectively; p = 0.72), or 75 h (20.0±0.44 vs. 19.3±0.40, respectively; p = 0.21). *, p<0.04.

Because of its utility in demonstrating the anticonvulsant effects of intermittent fasting, we also used the intraperitoneal kainic acid test, which potentially could mimic the effects of rapamycin [25]. However, the kainic acid test has a longer duration, such that comparisons with other acute seizures tests are only possible at the 6 h and 3 d time points. Although there were no differences in overall seizure scores (Fig. 4A), maximum seizure scores (Fig. 4B) or number of epochs spent in seizure stages ≥2 (Fig. 4C) at 6 h after rapamycin, rapamycin shortened the latency to stage 2 seizures, indicating that rapamycin may hasten the onset of seizures, but without an effect on seizure activity over the first two hours of kainic acid exposure (Fig. 4D). In contrast, mice treated with rapamycin for 3 consecutive days had lower overall seizure scores compared with vehicle, but only after 90 minutes of kainic acid exposure (Fig. 4E). Interestingly, overt seizure activity was not observed in rapamycin-treated mice for the last 3 time points in the study (Fig. 4E). However, there were no differences over the 2 h observation period in latency to stage 2 seizures, maximum seizure score, or number of epochs spent in seizure stages ≥2 (Fig. 4F–H). Body weights were similar between mice treated with rapamycin versus vehicle.

### Rapamycin does not protect in the PTZ test

**Figure 5:**
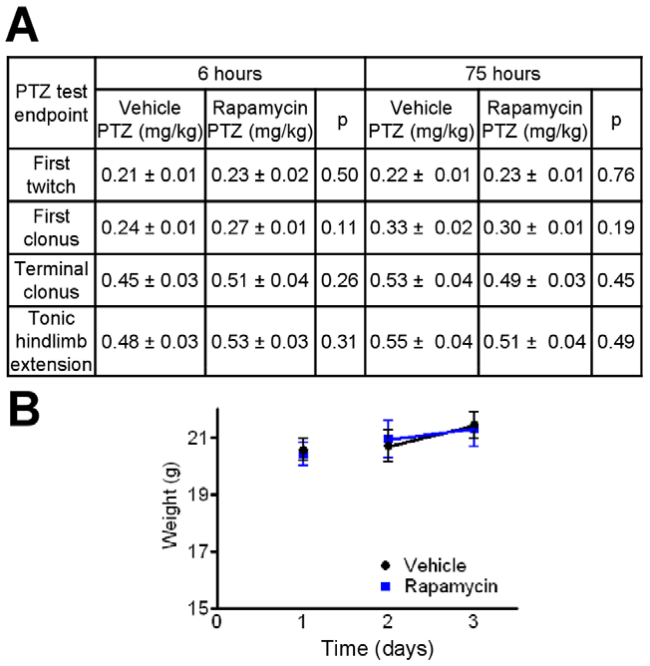
Rapamycin does not protect against i.v. PTZ-induced seizures. (A) Dose of PTZ (mean ±SEM) required for first twitch, first clonus, terminal clonus, and tonic hindlimb extension seizure behaviors in the PTZ test. Mice were tested in 3–5 independent experiments for both vehicle (N = 14–16) and rapamycin-treated mice (N = 17–18). Statistical comparisons were performed using a *t*-test. (B) Weights of all mice tested in PTZ test. Data points for the same animals weighed at different times are connected by lines. Each dose of rapamycin was 4.5 mg/kg.

Using the same 6 h and 3 d rapamycin treatment regimes as above, mice were not protected against PTZ-induced seizures for any of the seizure behaviors scored, including first twitch, initial clonus, terminal clonus, or tonic hindlimb extension (Fig. 5A). It is unlikely that a phenotype was missed because these data have a power of greater than 0.9 to detect a difference in latency to onset of clonus between treatment groups. Failure of rapamycin to protect mice in the PTZ test is not surprising as this test is not sensitive to other metabolism-based anticonvulsant treatments, including the ketogenic diet and intermittent fasting, despite utility of the PTZ test in rats consuming a ketogenic diet [25], [29], [30]. No differences in body weights were detected between mice treated with rapamycin versus vehicle in these cohorts (Fig. 5B).

### Rapamycin does not affect glucose and ketone levels

**Figure 6:**
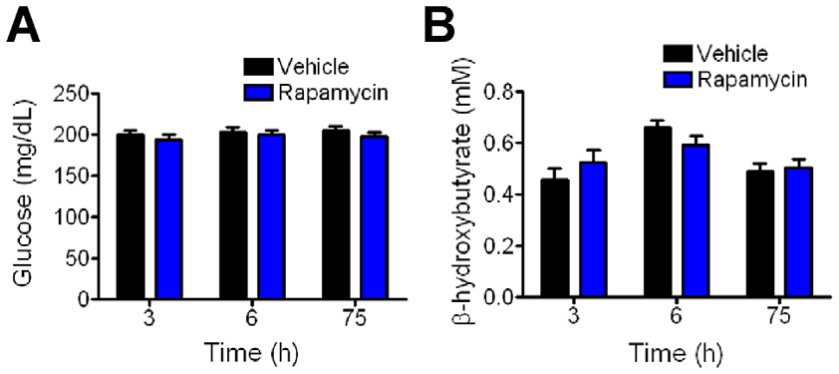
Glucose and β–hydroxybutyrate levels do not differ between mice treated with rapamycin and vehicle. (A) Blood glucose levels of rapamycin- and vehicle-treated animals at 3 h (N = 12 per group; p = 0.43, *t*-test), 6 h (N = 18 per group; p = 0.83, t-test) and 75 h (N = 32 per group; p = 0.33, *t*-test). (B) Blood levels of β-hydroxybutyrate for the same mice in panel A, 3 h (p = 0.3), 6 h (p = 0.13), 75 h (p = 0.68).

The mTOR pathway is sensitive to changes in glucose levels [7]. To determine whether seizure protection correlates with changes in systemic metabolism, we measured blood glucose and β-hydroxybutyrate levels prior to seizure testing. No differences were detected at the 3 h, 6 h, or 75 h time points (Fig. 6A & B). This suggests that the seizure protection observed here is not due to changes in levels of blood glucoses or β-hydroxybutyrate.

## Discussion

Although mTOR activity is suppressed by metabolism-based therapies that protect in acute seizure tests [31], [32], we show here that rapamycin has limited beneficial effects in preclinical acute seizure tests following short-term or longer term rapamycin exposure. The collective profile of acute seizure test results for rapamycin is also distinct from other metabolism-based therapies, including the ketogenic diet and intermittent fasting (which also differ from one another) (Table 1) [25]. Thus, no two types of metabolism-based therapies have been found to share the same acute seizure test profile, implying distinct mechanisms (Table 1). Even under conditions where rapamycin was protective, there were no changes in blood glucose or β-hydroxybutyrate levels, in contrast to other metabolism-based antiseizure treatments.

**Table 1:** Comparison of rapamycin to other antiseizure treatments.

	6 Hz	MES-T	PTZ	KA
Rapamycin (6 h)	0	+	0	0 or −
Rapamycin (3 d)	0	0	0	+
Ketogenic diet	+	0	0	−
Intermittent fasting (tested post-feed)	−	−	0	+
Phenytoin	0	+	0	+ or −
Lamotrigine	0	+	0	+
Topiramate	0	+	0	+

Protective (+), No effect (0), Lowers threshold (−).

### Rapamycin compared to other anticonvulsants

To provide potential insight into the mechanisms of rapamycin, we compared its acute seizure test profile with other anticonvulsant compounds. Protection by rapamycin against MES-T-induced seizures, albeit transient, combined with a lack of protective effects against PTZ- and 6 Hz-induced seizures is similar to the profile of three other anticonvulsants, specifically phenytoin, lamotrigine, and topiramate (Table 1) [33], [34], [35]. Thus, rapamycin exposure for ≤6 h has an anticonvulsant profile in mice that is most comparable to agents that suppress activity of voltage-dependent sodium channels. However, whole-cell patch clamp recordings with rapamycin in the presence of GABA_A_, AMPA, and NMDA receptor blockers showed no difference in current-voltage (I-V) relationships (vs. vehicle) [20]. Although these findings suggest that rapamycin does not directly affect sodium or potassium currents, this possibility was not further investigated with other pharmacological methods. Our data suggest the need for further study of the effect of rapamycin on sodium channels.

Rapamycin exhibits a dichotomous effect in the kainic acid test, as it protects only at the latest times after the onset of seizure activity (see Fig. 4). Whether the seizure test profile of rapamycin in the kainic acid test is shared with inhibitors of voltage-dependent sodium channel activity is less clear. Although kainic acid test results after short (i.e., <1 h) pretreatment with either phenytoin or lamotrigine are mixed [36], [37], topiramate protects against kainic acid-induced clonic and tonic seizures [38], while rapamycin only mildly suppressed the mean seizure score and only during the final 30 min of the test. Despite trends for the other scoring criteria, they were not significant despite the number of animals we tested. Topiramate also has inhibitory effects on AMPA/kainate-type ionotropic glutamate receptors and calcium channels and enhances GABA_A_ receptor function [39], [40], [41], [42]. The action of lamotrigine on N- and P-type calcium channels, unlike phenytoin and topiramate, could potentially explain differences in seizures profiles from rapamycin, although the mechanism of this effect remains unclear [43].

The use of seizure-naïve, non-epileptic mice in this study allowed us to examine the short-term effects of rapamycin on nonpathological tissue. This is in contrast to long-term effects in disease models that may be dependent on a specific pathological context (i.e., models of TSC). The use of seizure-naïve, non-epileptic mice is a strategy that has successfully identified a wide variety of anticonvulsants used in the clinic [22]. Although potentially useful in further exploring the antiseizure mechanisms of rapamycin, the use of normal mice prevents us from drawing conclusions about the effects of rapamycin on mice with epilepsy.

### Rapamycin compared to other treatments that affect metabolism

Similar to rapamycin, the high-fat, low-carbohydrate ketogenic diet suppresses mTOR activity [31]. The ketogenic diet has been suggested to decrease mTOR activity via increased AMPK activity [32]. If rapamycin and the ketogenic diet share similar metabolic effects and anticonvulsant mechanisms as suggested [31], then rapamycin treatment could be expected to protect against 6 Hz-induced acute seizures, similar to the ketogenic diet [28]. We found no such protection in the rapamycin dosing regimens studied here. To put these new findings in perspective with our ketogenic diet results, there was <1 mA difference in the mean CC_50_ between rapamycin- and vehicle-treated mice (Fig. 3A), in contrast to the 2 mA difference in CC_50_ between the ketogenic diet and normal diet [25]. Thus, despite similar effects on mTOR inhibition, rapamycin and the ketogenic diet appear unlikely to stop acutely-induced seizures (such as those used here) via the same mechanisms. With respect to recurrent seizures in chronic seizure models, rapamycin and a ketogenic diet both prevent seizures long after kainic acid-induced status epilepticus, but differ in their ability to prevent recurrent seizures after pilocarpine-induced status epilepticus [44], [45]. These differences do not rule out the possibility that rapamycin and the ketogenic diet share some long-term effects on recurrent seizures after status epilepticus.

AMPK activity is sensitive to multiple metabolic signals that ultimately affect ATP levels and in turn, increased AMPK activity inhibits mTOR activity [7]. Relevant to neuronal activity, AMPK-mediated changes in long-term potentiation are mTOR-dependent [46]. Differences in acute seizure test profiles between three different treatments that affect AMPK activity (i.e., rapamycin, ketogenic diet, and 2-deoxy-D-glucose) support the hypothesis that downstream effects of neuronal mTOR inhibition likely depend on additional factors specific to each intervention (for example, increased fatty acid concentrations that affect mitochondrial physiology).

### Potential mechanisms of rapamycin in seizure protection

Rapamycin is known to bind FKBP12 to specifically inhibit mTORC1activity. Evidence that rapamycin acts similarly *in vivo* is shown by the ability of rapamycin and its derivatives to decrease recurrent seizures in animals and patients where TORC1 activity is abnormally high. Thus, it generally is assumed that rapamycin exerts its antiseizure actions by decreasing TORC1 activity [9], [10], [11], [12]. Protection in drug-induced chronic seizure models raises the possibility that mTOR inhibitors reverse a seizure-induced increase in the mTOR pathway [15], [16]. Specifically, after kainic acid-induced status epilepticus, increases in mTOR activity (reflected in phosphorylation of S6) are noted 1–6 h after seizure onset, then decrease to baseline values, only to increase again 3 days after onset [15]. Both of these increases are reversed by administration of rapamycin [15]. However, the connection between mTOR activity and excessive neuronal activity during seizures is not clear. mTOR activity is required in dendrites for arbor and spine morphogenesis in some (but not all) studies, raising the possibility that these changes in neuronal morphology may impact seizures and/or epilepsy [20], [47], [48], [49], [50]. Rapamycin also inhibits mossy fiber sprouting in a number of models of status epilepticus [11], [15], [16]. However, the importance of inhibiting mossy fiber sprouting is unclear because rapamycin can prevent mossy fiber sprouting without protecting against seizures after pilocarpine-induced status epilepticus [19]. Electrophysiologically, mTOR is necessary for long-term potentiation [51], [52] and long-term depression [53], [54].The effect of rapamycin on synaptic transmission may be mediated via decreased neuronal excitability [21], [51], [55], [56] and/or neurotransmitter release [57]. Whether these morphological and physiological effects are the specific mechanism of seizure protection is unclear. In summary, decreased rapamycin-related neuronal excitability in some paradigms may be the result of mTOR inhibition but these studies do not rule out the possibility of an “off-target” (i.e., other than mTOR-related) effect, particularly given the broad effects of mTOR activity on protein synthesis, lipid metabolism, and autophagy [7].

The limited seizure protection after a 3 d rapamycin exposure in the seizure-naïve mice studied here may be due to unintended deleterious effects of prolonged mTOR suppression, in contrast to physiological mTOR suppressors where mTOR activity eventually rebounds [58]. Another potential explanation is that the 3 d rapamycin regimen used here may suppress activity of the other mTOR protein complex, TORC2, with a subsequent deleterious effect on Akt activity [59]. Consistent with only transient protection in the MES-T test, there may be an optimal degree of timing or extent of mTOR suppression that confers seizure protection in preclinical tests, though it is conceivably difficult to pharmacologically achieve such a balance. Finally, rapamycin is unlikely to have global antiseizure benefits, as it fails to protect in a model of infantile spasms induced by betamethasone and NMDA, even when administered before and after spasms started [60].

### Clinical implications

Pretreatment or sustained exposure to rapamycin (rather than post-seizure treatment with rapamycin) appears to be necessary to prevent seizures in preclinical models, as outlined previously. A requirement for prolonged rapamycin treatment is consistent with our finding that a 3-day treatment with rapamycin is more effective than a short 6 h treatment prior to kainic acid-induced seizures. Potentially more important than length of treatment, it appears that rapamycin is more effective in seizure models where mTOR activity is significantly increased at baseline, rather than situations where mTOR may be only transiently increased. This suggests the need for further study of rapamycin in situations where there is no known underlying mTOR-related pathology.
